# Cushing Syndrome: The Role of MSCs in Wound Healing, Immunosuppression, Comorbidities, and Antioxidant Imbalance

**DOI:** 10.3389/fcell.2019.00227

**Published:** 2019-10-09

**Authors:** Miriam Caffarini, Tatiana Armeni, Pamela Pellegrino, Laura Cianfruglia, Marianna Martino, Annamaria Offidani, Giovanni Di Benedetto, Giorgio Arnaldi, Anna Campanati, Monia Orciani

**Affiliations:** ^1^Department of Clinical and Molecular Sciences, Università Politecnica delle Marche, Ancona, Italy; ^2^Section of Biochemistry, Department of Clinical Sciences, Biology and Physics, Università Politecnica delle Marche, Ancona, Italy; ^3^Department of Experimental and Clinical Medicine, Clinic of Plastic and Reconstructive Surgery, Università Politecnica delle Marche, Ancona, Italy

**Keywords:** cushing syndrome, MSCs, wound healing, antioxidant capacity, immunosuppression

## Abstract

Cushing syndrome (CS), caused by glucocorticoid (GCs) excess, is strictly connected to onset of different metabolic diseases and impaired wound healing. The source of excessively high levels of GCs allows the identification of endogenous and exogenous (iatrogenic) CS. Iatrogenic patients usually receive also anti-metabolites serving as the foundation to modern steroid-sparing immunosuppressive therapy. Tissues mainly targeted by CS are bone and fat, both derived from progenitor cells named mesenchymal stem cells (MSCs). In addition, the pathogenic role of MSCs in other diseases sharing common properties with CS, such as an altered inflammatory profile and increased oxidative stress, has been identified. In this light, MSCs isolated from skin of control healthy subjects (C-MSCs), patients affected by endogenous CS (ENDO-MSCs), patients affected by iatrogenic CS (IATRO-MSCs) and patients affected by exogenous CS receiving steroid-sparing drugs (SS-MSCs), respectively, have been isolated and analyzed. ENDO- and IATRO-MSCs showed a reduced differentiative potential toward osteogenic and adipogenic lineages compared to C-MSCs, whereas SS-MSCs re-acquired the ability to differentiate, with a trend similar to control cells. In addition, MSCs from CS groups, compared to control MSCs, displayed a reduction in the secretion of cytokines (immune-suppression), a decreased expression of genes related to wound healing and a dysregulation of the enzymes/genes related to antioxidant capacity. In conclusion, our results suggest that the hallmarks of CS, such as wound healing impairment and immunosuppression, are already detectable in undifferentiated cells, which could be considered a potential therapeutic early target for control of CS.

## Introduction

Cushing syndrome (CS) is a clinical condition resulting from chronic exposure to excessively high levels of glucocorticoids (GCs) (endogenous or exogenous/iatrogenic). The most common form of CS is secondary to chronic treatment with GCs, commonly used for their anti-inflammatory action in many chronic immune-mediated and inflammatory clinical conditions. On the contrary, endogenous hypercortisolism is a rare disease (incidence 1.2−2.4 cases per million/year) dividing classically into two variants: ACTH-dependent (70%) and ACTH-independent (30%). The ACTH-dependent forms are characterized by the hypersecretion of ACTH by pituitary or, more rarely, extra-pituitary tumors. In ACTH-independent form, the cause is instead an adrenocortical tumor (adenoma and carcinoma) and less frequently a bilateral adrenal hyperplasia (Nieman et al., 2008; Arnaldi et al., 2012).

Glucocorticoids excess, whatever the etiology, determines multiple and complex consequences including obesity, hypertension, diabetes, thromboembolism, osteoporosis and fractures, myopathy, infections, skin alterations, and poor wound healing (Arnaldi et al., 2003). Care and control of all comorbidities should be one of the primary goals during the diagnosis and long-term follow-up of these patients.

Moreover, the modern therapeutic management of patients suffering from inflammatory and/or immune-mediated diseases, is usually based on therapeutic combination of corticosteroids and steroid sparing agents (methotrexate, mycophenolate mofetil, and azathioprine), able to reduce the dose of GCs to be administered to the patient to control the disease and therefore hinder the emergence of exogenous Cushing.

Tissues mainly damaged in CS include fat and bone (Feldman, 2009), both derived from progenitor cells named MSCs that, in this light, may be in turn affected by glucocorticoid excess too. In addition, patients with CS usually show skin atrophy, difficulty in wound healing and tendency to the formation of bruises even for minimal/unapparent trauma. Skin fibroblasts of patients affected by CS are characterized by early degenerative phenomena leading to atrophy and wound healing impairment, both in the forms of exogenous and endogenous CS. Detrimental effects on wound healing may be due also to an excessive ROS production, caused by prolonged levels of elevated GCs (Dunnill et al., 2017). ROS act in the recruitment of lymphoid cells to the wound site and effective tissue repair, when angiogenesis occurs and in the creation of a microenvironment with bacteriostatic effects (Dunnill et al., 2017); on the other hand, excessive production of ROS or impaired ROS detoxification causes oxidative damage, which is the main cause of non-healing chronic wounds (Cano Sanchez et al., 2018).

In other skin pathologies characterized by an altered inflammatory profile and increased oxidative stress, such as psoriasis and atopic dermatitis, the involvement of MSCs has been already investigated, and recognized (Campanati et al., 2012, 2017, 2018a). To date, no indications are still available about the behavior of MSCs derived from skin of patients affected by endogenous and iatrogenic (exogenous) CS; this works aims to isolate MSCs from these cohorts of subjects and evaluate their differentiative potential, wound healing and antioxidant capacity.

## Materials and Methods

### Ethics Statement

All patients provided their written informed consent to participate to the study, which was approved by the institutional ethics committees and was conducted in accordance with the Declaration of Helsinki.

### Human Tissue Collection

For MSCs isolation, 12 patients were included into the study and divided in four groups: 3 controls (C-MSCs, healthy subjects), 3 endogenous (ENDO-MSCs, patients affected by pituitary CS), 3 iatrogenic (IATRO-MSCs, patients affected by exogenous CS), 3 steroid-sparing (SS-MSCs, patients affected by exogenous CS receiving steroid-sparing drugs). Diagnosis of exogenous CS was clinically made according to the presence of traditional stigmata as follows: weight gain, usually presenting as central obesity with redistribution of body fat to truncal areas and the appearance of dorsocervical and supraclavicular fat pads and the classic moon face; plethora, easy bruising, thin skin, striae, myopathy, and muscle weakness (particularly proximal muscles), susceptibility to poor wound healing and increased incidence of infection. Patients with exogenous CS were receiving systemic steroids for therapeutic control of bullous skin diseases (pemphigus and pemphygoid) in monotherapy or in association with steroid sparing agents, and they were all in complete remission for skin bullous diseases signs and symptoms.

Diagnosis of endogenous CS due to ACTH-secreting pituitary tumor was made by a trained endocrinologist, according to the consensus statement and clinical practice guidelines (Nieman et al., 2008; Arnaldi et al., 2012).

Demographic and clinical data of enrolled patients are reported in Table 1.

**TABLE 1:** Demographic and treatment profile of enrolled patients.

**Patients**	**Age**	**Sex**	**Group**	**24 h Urinary cortisol times × ULN**	**Disease duration (months)**
1	59	F	C-MSCs		
2	63	F	C-MSCs		
3	67	M	C-MSCs		
4	51	F	ENDO-MSCs	**4.5**	**36**
5	49	F	ENDO-MSCs	**4**	**30**
6	33	F	ENDO-MSCs	**4**	**12**
				**Type, dose and duration of steroid treatment**	
7	65	F	IATRO-MSCs	Oral Prednisone 25 mg/daily – 24 weeks	
8	58	F	IATRO-MSCs	Oral Prednisone 25 mg/daily – 24 weeks	
9	64	M	IATRO-MSCs	Oral Methylprednisolone 16 mg/daily – 36 weeks	
					**Type, dose and duration of steroid sparing treatment**
10	65	F	SS-MSCs	Oral Prednisone 12,5 mg/daily – 24 weeks	Oral Azathioprine 100 mg/daily − 24 weeks
11	69	F	SS-MSCs	Oral Prednisone 10 mg/daily – 24 weeks	Oral Azathioprine 150 mg/daily − 24 weeks
12	59	M	SS-MSCs	Oral Methylprednisolone 8 mg/daily – 36 weeks	Oral Mycophenolate sodium1440 mg/daily – 36 weeks

**ULN, upper limit of the normal range.**

All subjects underwent a skin punch biopsy, which was taken from the extensor surface of left arm with a 6 mm sterile cutaneous skin punch biopsy device (Gima, medical devices, s.r.l. Rome, Italy), after administrating local anesthesia with 2% lidocain.

### Cell Culture

As previously described (Orciani et al., 2011, 2017; Campanati et al., 2018b), tissue fragments (2–3 mm^3^) were subjected to mechanical digestion, placed into 6-well plates containing MSCGM (Euroclone, Milan, Italy) medium – to enhance the growth of undifferentiated cells – and then maintained in culture by using the same medium at 37°C in 95% air −5% CO_2_. The growth medium was changed after 24 h to remove unattached cells and then replaced with fresh medium twice a week. Cell morphology was evaluated by phase-contrast microscopy (Leica DM IL; Leica Microsystems GmbH, Wetzlar, Germany) and viability was analyzed by an automated cell counter (Invitrogen, Milano, Italy). All further analyses involved separate assays of the specimens from each participant up to the first five passages.

### Characterization of MSCs

According to the criteria identified by Dominici et al. (2006), cells were characterized by testing their plastic adherence, the immunophenotype and the multipotency.

For immunophenotyping, 2.5×10^5^ cells were stained for 45 min with fluorescein isothiocyanate (FITC)-conjugated antibodies (Becton-Dickinson) against: HLA-DR, CD14, CD19, CD34, CD45, CD73, CD90, and CD105.

For differentiation assay, cells were induced toward osteocytes and adipocytes using STEMPRO^®^ Osteogenesis and Adipogenesis Kits (GIBCO, Invitrogen), respectively (Orciani et al., 2013). Osteogenic differentiation was assessed by Alizarin Red staining after 10 days of induction; adipogenic differentiation was tested by Oil Red staining after 15 days of induction. Cells cultured in MSCGM alone were used as negative controls. For the quantification, Alizarin Red was detached by incubating with 10% cetylpyridinium chloride for 30 min at RT, then optical density was measured and quantified through a plate reader (Multiskan GO microplate reader, Thermo Fisher Scientific).

### ELISA of Inflammation-Related Cytokines

Selected cytokines related to inflammation, IL1-α, IL1-β, IL2, IL4, IL6, IL8, IL10, IL12, IL17A, IFN-γ, and G-CSF were investigated by ELISA (Multi-Analyte ELISArray kit, Qiagen, Milan, Italy) as previously described (Campanati et al., 2017). Briefly, medium conditioned for 72 h by each sample of MSCs (1 × 10^5^ cells at passage 5th) was used for the test. Samples were dispensed into a 96-well microtiter plate and incubated for 2 h at room temperature. After washing, avidin-HRP-conjugated antibody was added to the plate and incubated for 30 min. Finally, captured cytokines were detected by addition of substrate solution. The OD at 450 nm was determined using a microtiter plate reader.

The level of each cytokine detected in CS groups was calculated as % of its level detected in C-MSCs; subsequently, mean ± SD from three independent experiments was calculated.

### RT-PCR Analysis of the Expression of Selected Genes

The expression of genes related to wound healing (EGF, FGF, PDGF, and VEGF), to antioxidant capacity (GCLC, GSTA1, GSTA2, GSTM1, GPX1, CAT, and GR) and nuclear factor kappa-light-chain-enhancer of activated B cells (NF-κB) was analyzed by Real Time PCR (RT-PCR); total RNA was isolated from 1 × 10^6^ cells at passage 4 by using 5 PRIME PerfectPure RNA Purification (5 PRIME, Hamburg, Germany) and retrotranscribed to cDNA (GoScript^TM^ Reverse Transcription System, Promega, Italy). All samples were tested in triplicate with the housekeeping genes RPLP0 and GAPDH for data normalization. Of these two, GAPDH was the most stable one and was used for subsequent normalization. After amplification, melting curves were acquired. Direct detection of PCR products was monitored by measuring the fluorescence produced by SYBR Green I dye (EVA Green PCR Master Mix, Bio-rad) binding to double strand DNA after every cycle. These measurements were then plotted against cycle numbers. The parameter threshold cycle (Ct) was defined as the cycle number at which the first detectable increase above the threshold in fluorescence was observed.

The amount of mRNA detected in SC patients was calculated as X-fold respect to C-MSCs (expressed as 1) by the 2^–ΔΔ*Ct*^ method (Lazzarini et al., 2014), where ΔCt = Ct (gene of interest) – Ct (control gene) and Δ (ΔCt) = ΔCt (ENDO-, or IATRO-, or SS-MSCs) – ΔCt (C- MSCs). X-fold was calculated for the selected genes in all the twelve samples of MSCs. Subsequently, mean ± SD from three independent experiments in triplicates was calculated and displayed. All the primer sequences are reported in Table 2.

**TABLE 2:** Sequence of the primers used in Real Time PCR.

**Gene Symbol**	**Forward**	**Reverse**
GAPDH	5′-AGCCACATCGCTCAG ACAC-3′	5′-GCCCAATACGACC AAATCC-3′
RPLPO	5′-CCATTCTATC ATCAACGGGTACAA-3′	5′-TCAGCAAGTGGGA AGGTGTAATC-3′
VEGF	5′-CCTCCGAAACCATGA ACTTT-3′	5′-ATGATTCTGC CCTCCTCC TTCT-3′
FGF	5′-AGTCTTCGCCAGGTC ATTGA-3′	5′-CCTGAGTATTCGGCA ACAGC-3′
PDGF	5′-TGGAAGTGCAGAG GTCTCAG-3′	5′-GCGAGGAGGTGTG GTTTCTA-3′
GCLC	5′-GGAAGTGGATGTGGA CACCAGA-3′	5′-GCTTGTAGTCAGGAT GGTTTGCG-3′
GSTA1	5′-GCAGACCAGAGCCATT CTCAAC-3′	5′-ACATACGGGCAGAAG GAGGATC-3′
GSTA2	5′-CTGCCCTTTAGTCAAC CTGAGG-3′	5′-ACAAGGTAGTCTTGTC CGTGGC-3′
GSTM1	5′-TGATGTCCTTGACCTC CACCGT-3′	5′-GCTGGACTTCATGTA GGCAGAG-3′
GPx1	5′-GTGCTCGGCTTCCC GTGCAAC-3′	5′-CTCGAAGAGCATGAA GTTGGGC-3′
CAT	5′-GTGCGGAGATTCAAC ACTGCCA-3′	5′-CGGCAATGTTCTCACA CAGACG-3′
GR	5′-CCTACCCTGGTGTC ACTGTT-3′	5′-CCTTTGCCCATTTC ACTGCT-3′
NF-κB	5′-AATGGTGGAGTCTG GGAAGG-3′	5′-TCTGACGTTTCC TCTGCACT-3′

### Preparation of Cellular Extracts

Cultured cells were resuspended in phosphate buffered saline (PBS) containing aprotinin (1 μg/ml), centrifuged at 500 × *g* for 5 min, at 4°C, and finally lysed with 10 mM sodium phosphate, pH 6.0, containing 0.5% v/v Non-idet P40, at 4°C. After 30 min incubation on ice, cell lysates were centrifuged at 13000 × *g* for 15 min, at 4°C. Supernatants were then collected and the activities of the antioxidant enzymes (CAT, GST, GR, Se-dependent and Se-independent GPX) were analyzed. Total protein concentration was determined by the Bradford protein assay.

### Quantitative Determination of Total Glutathione

The levels of total glutathione (GSH + GSSG) were measured in MSCs suspended in 100 μl PBS, deproteinized in 5% sulfosalicylic acid and 4 mM EDTA. The samples were incubated for 30 min at 4°C and centrifuged at 2300 × *g*, for 2 min. Supernatants were recovered and assayed spectrophotometrically (at 412 nm) by using the glutathione reductase (GR) recycling assay in the presence of 5,5’-dithiobis (2-nitrobenzoic acid) (DTNB), with a calibration line based upon known concentrations of GSH (Brigelius et al., 1983). To prevent GSH artificial oxidation during sample processing, cells were washed twice (1 min each) at room temperature with PBS containing 5 mM N-ethylmaleimide (NEM) (Sigma-Aldrich, Milan, Italy), according to an optimized protocol for the reliable measurement of GSH, GSSG, and PSSG in cell cultures (Giustarini et al., 2015). The pellet was resuspended with 1 M NaOH for the quantification of proteins. The level of total glutathione was expressed as nmol/mg protein.

### Enzymatic Activity Assays

Glutathione reductase (GR) activity was measured using the method described by Carlberg and Mannervik (1975). The assay evaluates the decrease in absorbance at 340 nm due to NADPH oxidation during the reduction of GSSG (ε = −6.22 mM^–1^ × cm^–1^). The assay was carried out in 100 mM sodium phosphate, pH 7.0, 0.1 mM NADPH and 1 mM GSSG. The activity of GR was calculated by using an extinction coefficient for NADPH of 6.22 mM^–1^ × cm^–1^ and the results were expressed as nmol of NADP^+^ per min per mg of proteins.

Glutathione-S-transferase GST) activity was measured according to the method described by Habig et al. (1974), i.e., 1-chloro-2,4-dinitrobenzene (CDNB) was used as substrate and absorbance of resulting products was measured at 340 nm. Specifically, this colorimetric assay is based upon the GST-catalyzed reaction between GSH and the GST substrate, CDNB, which has the broadest range of isozyme detectability (e.g., alpha-, mu-, pi-, and other GST isoforms). The assay was carried out in 100 mM sodium phosphate, pH 6.5, 1 mM CDNB and 1 mM GSH. GST activity (defined as the amount of enzyme producing 1 μmol of CDNB-GSH conjugate/min under the conditions of the assay) was calculated using an extinction coefficient for CDNB of 9.6 mM^–1^ × cm^–1^. Results were expressed as μmol of CDNB-GSH conjugates per min per mg of proteins.

Glutathione peroxidases (GPx’s) activity was assayed in a coupled enzyme system, where NADPH is consumed by glutathione reductase to convert the formed GSSG into its reduced form (GSH) (Brigelius-Flohe and Maiorino, 2013). The decrease of absorbance was monitored at 340 nm (ε = 6.22 mM^–1^ × cm^–1^) by using 0.8 mM cumene hydroperoxide as substrate for the Se-dependent GPx and for the sum of Se-dependent and Se-independent enzyme forms. A final volume of 1 ml contained 100 mM potassium phosphate buffer, pH 7.5, 1 mM EDTA, 1 mM sodium azide (NaN_3_ for the hydrogen peroxide assay), 2 mM GSH, 0.24 mM NADPH, 1 unit of GR and 0.5 mM H_2_O_2_ or 0.8 mM cumene hydroperoxide, as substrate.

Catalase (CAT) activity was measured by the decrease in absorbance at 240 nm (ε = 0.04 mM^–1^cm^–1^) due to the consumption of hydrogen peroxide, H_2_O_2_ (12 mM H_2_O_2_ in 100 mM potassium phosphate, pH 7.0).

### Statistical Analysis

Statistical analysis of data obtained from at least 3 independent experiments was performed by means of SPSS 19.0 software (SPSS, Inc., Chicago, IL, United States). All data are reported as mean ± SD.

Statistical analyses included the ordinary one-way ANOVA test for multiple comparison, and a *p*-value less than 0.05 was considered statistically significant.

## Results

Mesenchymal stem cells were successfully isolated from all the 12 patients, sub-grouped into “controls” (C-MSCs), “endogenous” CS (ENDO-MSCs), “iatrogenic” CS (IATRO-MSCs) and “steroid-sparing” CS (SS-MSCs). No statistically relevant difference was found in each cellular group among the donors. Therefore, results are reported as mean ± SD for C-, ENDO-, IATRO- and SS-MSCs in each analysis. For all the subsequent experiments, MSCs were used at the same culture passage.

### Cell Isolation and Characterization

**FIGURE 1:**
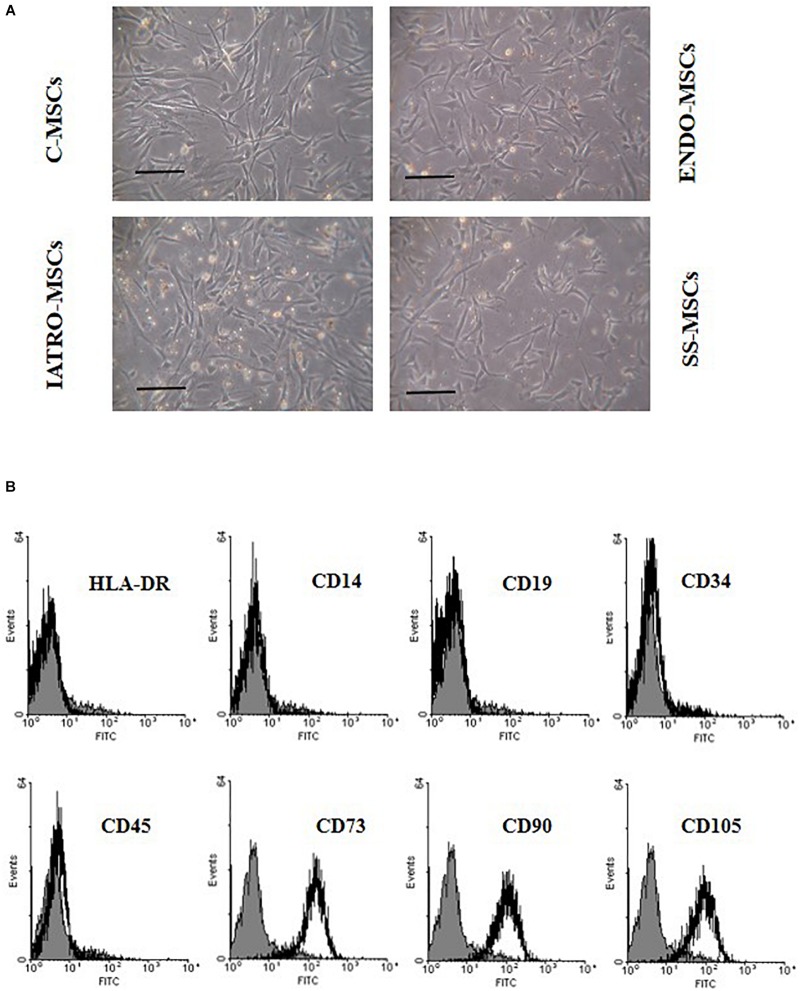
Cell morphology and immunophenotype of MSCs. **(A)** Phase-contrast images of MSCs derived from skin of control subjects (C-MSCs) and from skin of patients affected by endogenous cushing syndrome (CS) (ENDO-MSCs), iatrogenic CS (IATRO-MSCs), and iatrogenic CS under treatment with steroid sparing (SS-MSCs) Scale bar = 100 μm. **(B)** Representative FACScan analyses of cell-surface antigen expression, as indicated. Solid gray histograms refer to the negative control (IgG1 isotype control-FITC labeled). No differences were observed between MSCs isolated from the different subgroups.

Cell cultures from the 12 patients showed a fibroblast-like morphology (Figure 1A).

Isolated cells were plastic adherent in culture, strongly positive for CD73, CD90, and CD105 and negative for HLA-DR, CD14, CD19, CD34, and CD45 (Figure 1B) without significant differences among the subgroups.

**FIGURE 2:**
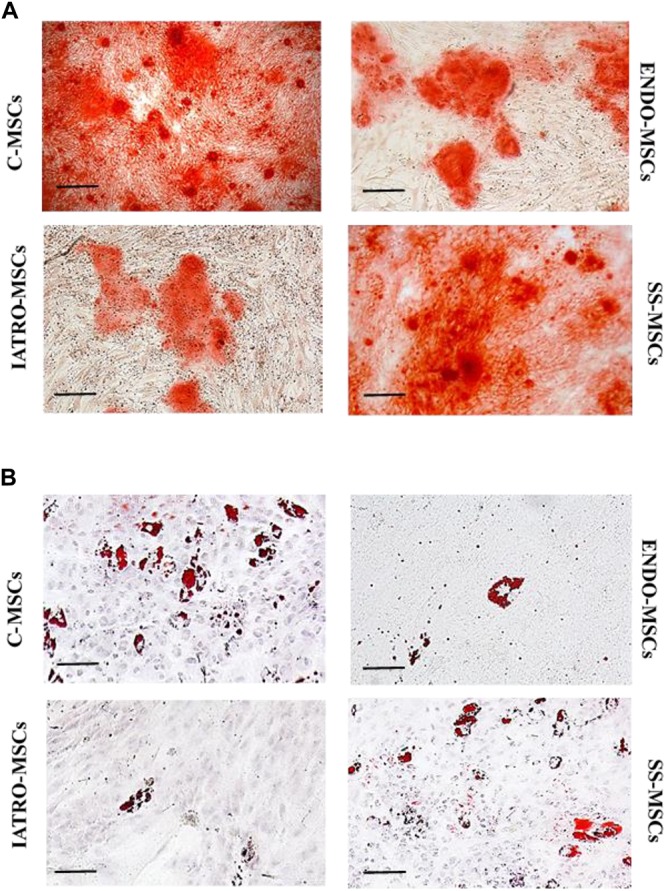
Multilineage differentiation of MSCs. Representative images of differentiation experiments. **(A)** Osteogenic differentiation after staining with Alizarin Red. **(B)** Adipogenic differentiation by Oil Red staining. Scale bar = 100 μm.

When cultured with specific supplements, C-MSCs were highly able to differentiate in osteoblasts and adipocytes, whereas MSCs belonging to ENDO- and IATRO- subgroups showed a very limited differentiative potential. The administration of steroid sparing agents contributed to preserve the differentiating ability of MSCs toward adipocyte and osteocyte lineages (Figure 2).

**FIGURE 3:**
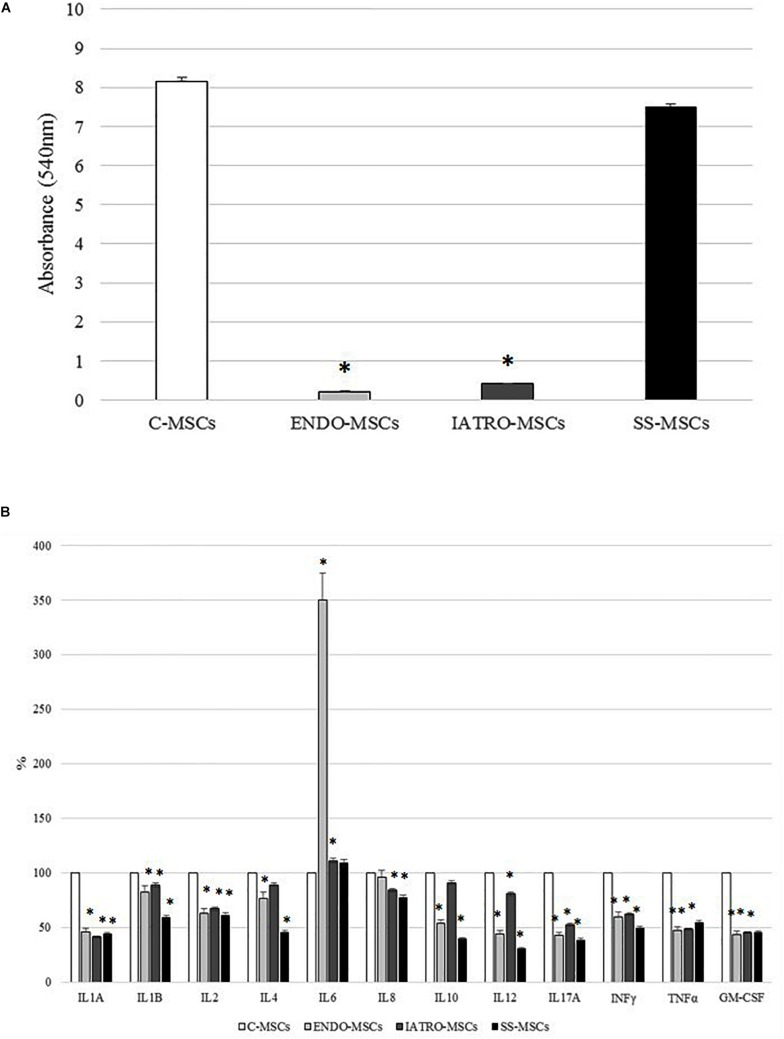
Alizarin Red staining quantification and secretion of cytokines. **(A)** Quantification of the Alizarin Red staining by cetylpyridinium chloride. **(B)** Secretion of cytokines related to inflammation by ELISA test. The levels measured in C-MSCs were considered as 100% and those detected in MSCs derived from SC patients accordingly calculated; ^∗^*p* < 0.05 MSCs from SC patients vs. C-MSCs.

The quantification of the Alizarin Red staining by cetylpyridinium chloride confirmed that C- and SS-MSCs were more able to differentiate toward osteoblasts than ENDO- and IATRO-MSCs (Figure 3A).

### Expression Profile of Inflammatory Cytokines

The secretion of several cytokines related to inflammation was evaluated by ELISA.

In general, the level of secreted cytokines was lower in MSCs derived from CS patients [both affected by endogenous and exogenous CS] and SS-MSCs than in C-MSCs (Figure 3B). In detail, the decrease respect to C-MSCs was always statistically significant except for IL4 and IL10 detected in IATRO-MSCs and IL8 in ENDO-MSCs. Notably, the expression of IL6 was higher in ENDO- and IATRO-MSCs than in C- and SS-MSCs.

The clinical use of steroid sparing agents does not produce any effect on the levels of secreted cytokines from MSCs.

### Gene Expression

The expression of selected genes referred to wound healing (FGF, PDGF, and VEGF) was analyzed by RT-PCR in MSCs derived from control subjects and from patients affected by endogenous and exogenous CS (both treated and untreated with SS).

**FIGURE 4:**
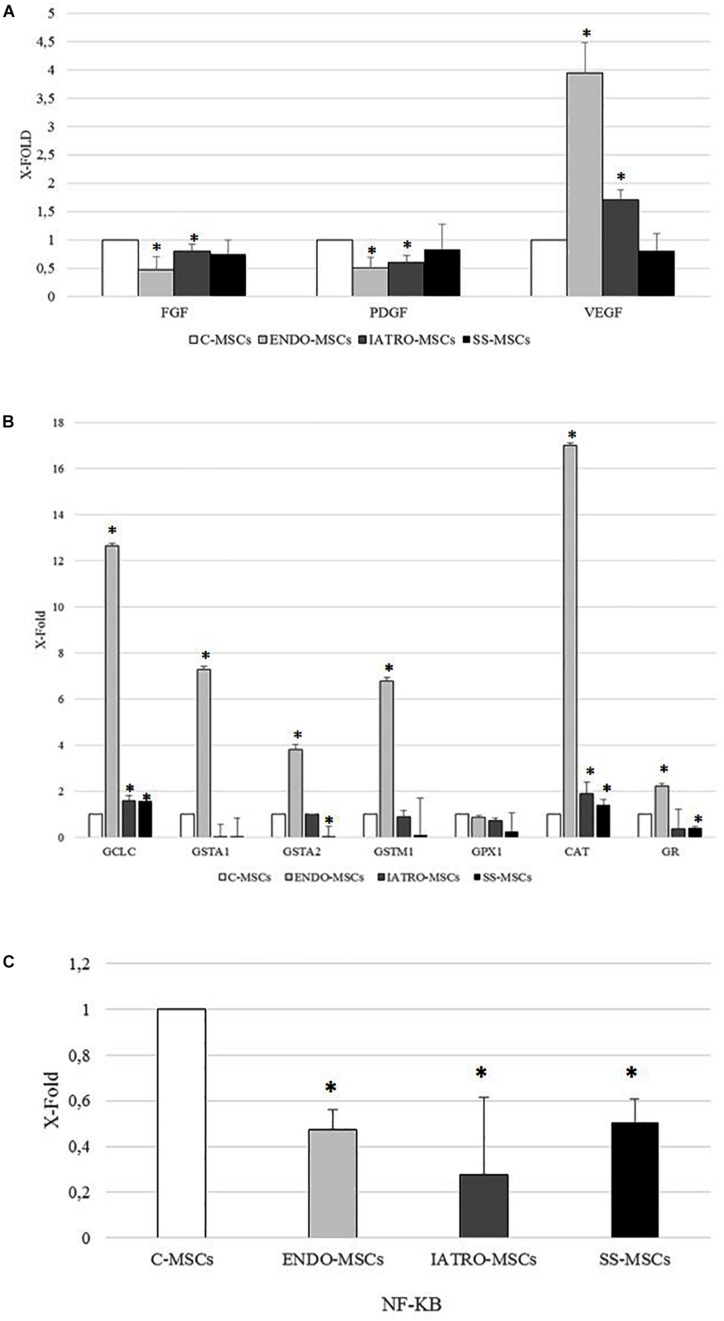
Analysis of the expression of selected genes by RT-PCR. The expression levels measured in MSCs from SC groups are considered as X-fold with respect to C-MSCs (referred as 1). Data are mean ± SD of analyses performed in three different cultures of each group, upon three independent experiments in triplicates. ^∗^*p* < 0.05 MSCs from CS groups vs. C-MSCs. **(A)** PCR analysis of genes referred to wound healing (FGF: Fibroblast Growth Factor; PDGF: Platelet Derived Growth Factor; VEGF: Vascular Endothelial Growth Factor). **(B)** PCR analysis of genes referred to antioxidant capacity (GCLC: Glutamate-Cysteine Ligase Catalytic Subunit; GSTA1: Glutathione S-transferase A1; GSTA2: Glutathione S-transferase A2; GSTM1: Glutatione S-transferase mu; GPX1: Glutathione peroxidase 1; CAT: catalase; GR: Glutathione Reductase). **(C)** PCR analysis of nuclear factor kappa-light-chain-enhancer of activated B cells (NF-KB).

FGF and PDGF genes were lower in CS MSC group compared to C-MSCs. The expression of VEGF was increased in IATRO- and even more in ENDO-MSCs compared to C-MSCs; steroid sparing allowed to maintain conditions resembling those of the control cells (Figure 4A).

As far as genes related to antioxidant capacity are concerned, the expression of all selected genes was higher in MSCs derived from skin of patients affected by endogenous CS than in the other cellular groups. A significant increase, even if weaker than in ENDO-MSCs, was observed also in the expression of CAT and GCLC of ESO- and SS-MSCs, when compared to the C-MSCs. On the contrary, SS-MSCs showed a reduced expression of GSTA2 and GR (Figure 4B).

Finally, the expression of NF-B was lower in MSCs from all patients affected by CS than in MSCs derived from control subjects (Figure 4C).

### Total Glutathione Levels Detection

**FIGURE 5:**
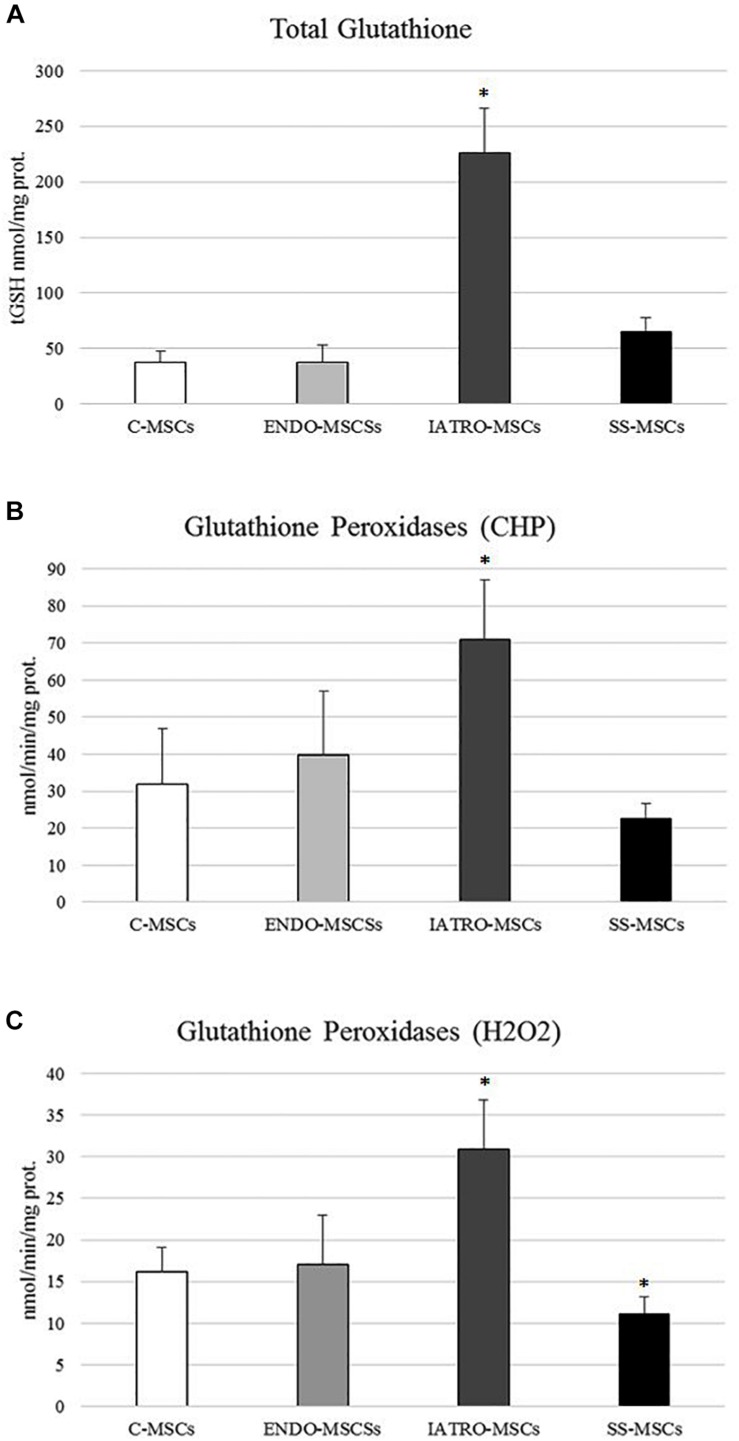
**(A)** Amounts of total glutathione (GSH and GSSG in GSH equivalent) expressed as nmol/mg protein. **(B)** Glutathione Peroxidase activity was measured for both Se-dependent enzyme (H_2_O_2_) and total GPX (CHP) activity. **(C)** Results are reported as mean values ± SD of three independent experiments. ^∗^*p* < 0.05 MSCs from CS patients vs. C-MSCs.

In order to investigate the cellular redox homeostasis in patients affected by CS, the putative involvement of glutathione (GSH), the most abundant cellular antioxidant and major modulator of the intracellular redox status, was evaluated (Figure 5A). GSH levels were 10-fold higher in patients affected by exogenous CS in comparison to C-MSCs and ENDO-MSCs, whereas steroid sparing treatment lead the GSH level near to the control.

### Glutathione Dependent Enzymes Analysis

In order to assess whether GSH-dependent antioxidant enzymes were affected by CS condition, the activity of glutathione S-transferases (GSTs), glutathione peroxidases (GPXs) and glutathione reductase (GR), as well as of CAT were evaluated in MSCs derived from the four groups.

In general, all the enzymes showed the same trend: MSCs isolated from patients affected by endogenous CS reached the highest values whereas the treatments with the SS restored conditions more similar to those observed in control cells. ENDO-MSCs displayed no significant difference in enzymatic activity when compared to C-MSCs.

In detail, GPXs activity was evaluated in cells through an assay able to detect both Se-dependent and Se-independent isoenzymes (CHP) and an assay able to selectively detect only Se-dependent form. Compared to C-MSC, a significant increase in both GPXs activities was observed in ENDO-MSCs, whereas steroid sparing treatment caused a strong reduction (Figures 5B,C). No significant variations were detected in ENDO-MSCs. These results indicate that a clear correlation between GPXs activity and GSH synthesis exists, since both analyses revealed the same trend.

**FIGURE 6:**
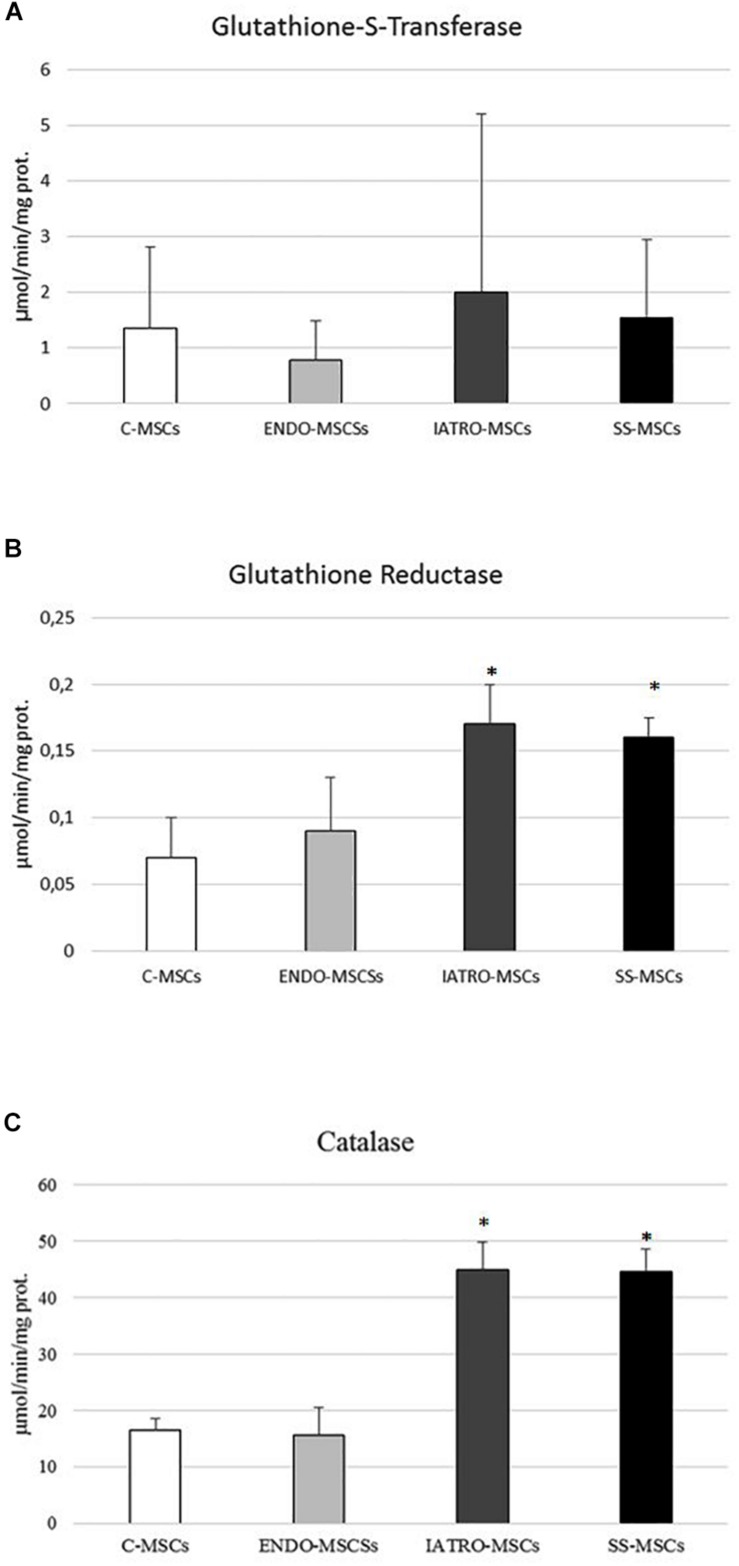
**(A)** Glutathione-S-transferase (GST), **(B)** glutathione reductase (GR), and **(C)** catalase (CAT) activity was measured by specific enzymatic assays. Values are reported as mean ± SD of three independent experiments. ^∗^*p* ≤ 0.05 MSCs from CS patients vs. C-MSCs.

Glutathione S-transferases analysis showed a significant increased activity in IATRO-MSCs compared to C-MSCs and ENDO-MSCs; this change was completely reset by SS treatment (Figure 6A).

GR activity and CAT shared the same trend: IATRO-MSCs reached the highest values, followed by ENDO- and C-MSCs. The use of SS, in these cases, did not convey to more physiological conditions (Figures 6B,C).

## Discussion

Cushing syndrome, caused by glucocorticoid excess, is strictly connected to the onset of different metabolic complications and impaired wound healing (Gordon et al., 1994).

The main tissue targets in course of CS are fat and bones (Feldman, 2009): in these tissues, GCs promote adipogenesis and inhibit osteogenesis by reducing c-Jun expression and bone marrow stromal cells proliferation. As these tissues share a common progenitor that is the MSC it may be postulated that CS could early affects the properties of MSCs.

In this scenario, the potential early involvement of MSCs in endogenous and exogenous CS has been investigated. MSC have been isolated from skin of control healthy subjects (C-MSCs), patients affected by endogenous (ENDO-MSCs) or iatrogenic CS (IATRO-MSCs) and patients affected by iatrogenic CS, treated with steroid combined with steroid sparing agents (SS-MSCs). MSCs were characterized according to the criteria defined by Dominici et al. (2006); isolated cells satisfied the three main criteria: they were plastic adherent, strongly positive for CD73, CD90, CD105 (negative for HLA-DR, CD14, CD19, CD34, and CD45) and able to differentiate toward osteogenic and adipogenic lineages.

One of the most important clinical consequence in course of all forms of CS is the wound healing impairment, a complex mechanism that involves different cell types, growth factors and several cytokines. It is well accepted that GCs are involved in the impairment of wound healing since GCs act by trans-repressing the pro-inflammatory cytokines and growth factors (Schacke et al., 2002; Slominski and Zmijewski, 2017) and by increasing the production of ROS (Bjelaković et al., 2007) which lead to oxidative stress and may have detrimental effects on wound healing (Rodriguez et al., 2008; Schafer and Werner, 2008; Ponugoti et al., 2013; Dunnill et al., 2017).

In this light, growth factors and secretion of soluble factors related to inflammation and wound healing, as well as antioxidant capacity, were evaluated and compared between MSCs derived from skin of controls and CS patients, respectively.

Firstly, the expression of PDGF and FGF was lower in MSCs derived from CS patients than in MSCs obtained from controls, whereas VEGF displayed an opposite trend. These results are in line with those obtained from others: Beer et al. (2000) reported a decrease in the expression of PDGF in wound healing of glucocorticoid-treated mice. Moreover, it has been observed a FGF reduction in skin wounds of glucocorticoid-treated animals, which showed a delay in tissue repair that was reversible after exogenous application of FGF (Brauchle et al., 1995). The decreased levels of FGF and PDGF detected in MSCs from CS patients compared to controls correlateed with the typical impairment of wound healing. Notably, VEGF was more expressed by MSCs obtained from CS patients, enforcing previous data reporting higher VEGF circulating levels in patients with Cushing’s syndrome than those detected not only in healthy subjects, but also in patients with primary aldosteronism and essential hypertension (Zacharieva et al., 2004, 2005).

The expression of these genes by SS-MSCs was not significantly different from that detected in C-MSCs. IL1α, IL1β, and TNF-α are strongly up-regulated during the inflammatory phase of healing (Grellner et al., 2000) and their expression is essential for normal tissue repair: previous works reported that the expression of these molecules was strongly reduced after wounding of healing-impaired glucocorticoid-treated mice (Hübner et al., 1996; Beer et al., 2000). Our results enforce these previous observations: the level of IL1α, IL1β, and TNF-α were lower in MSCs from CS groups than in C-MSCs, thus indicating a potential failure in the inflammatory phase of healing. More in general, it is well known that GCs suppress cell-mediated immunity by inhibiting genes that code for IL1, IL2, IL3, IL4, IL5, IL10, IL12, GM-CSF, and IFN-γ (Brattsand and Linden, 1996; Leung and Bloom, 2003). MSCs isolated from skin of CS patients showed a similar profile: the expression of IL2, IL4, IL10, IL12, GM-CSF, and IFN-γ was lower when compared to MSCs from control subjects. Treatment with steroid sparing agents did not produce effects on the secretion of the analyzed cytokines.

Particularly noteworthy, the expression of IL6 was increased in MSCs from patients with CS, especially in patients with endogenous CS, where the highest levels were found. Our findings are in agreement with previous studies reporting that circulating levels of IL6 are high in patients with overt hypercortisolism (Paoletta et al., 2011). This inflammation marker is associated with endothelial dysfunction and increased cardiovascular risk. Moreover, a direct relationship has been observed between IL6 in fatty tissue and insulin resistance in human obesity. Our results confirm that inappropriately high levels of IL6 in MSCs of CS patients during cutaneous repair may represent an important pathogenic component of the inflammatory-related vascular and metabolic complications associated with glucocorticoid excess (Barahona et al., 2009).

Glucocorticoid-induced osteoporosis is the leading form of secondary osteoporosis; GCs affect bone through immediate and sustained decrease of bone formation together with early and transient increase of bone resorption (Trementino et al., 2014; Minetto et al., 2019). MSCs from CS patients showed a reduced ability to differentiate into osteoblasts and this might reflect the observed decrease of bone formation. Notably, MSCs from patients treated with steroid sparing agents maintained a strong ability toward osteogenic differentiation. In addition, the mechanisms driving bone resorption induced by GCs are still unclear and contradictory (La Noce et al., 2019); among the others, IL6 has been suggested to be involved since it is renowned as a potent osteoclastogenic cytokine, involved indeed in the pathological bone resorption occurring in several bone diseases. Previous studies demonstrated that patients treated with high-dose of GCs had an enhancement of IL6 and IL6-dependent osteoclastogenesis (Dovio et al., 2006). Our data are in line with these observations: the level of secreted IL6 is very notably higher in MSCs from endogenous CS than in other cell subgroups. Surprisingly, even if excess of GCs has been often linked to increased adipogenesis (Lee et al., 2014), MSCs from CS patients show detrimental ability to differentiate into adipocytes. Previous studies report that GCs are required for full differentiation of adipocytes (Chapman et al., 1985), but they focus on preadipocytes that are already committed cells, unlike MSCs.

The major mechanism for this immunosuppression is through inhibition of NF-κB. NF-κB is a critical transcription factor involved in synthesis of many mediators (i.e., cytokines) and proteins (i.e., adhesion proteins) that promotes the immune response, thus it was not surprising that its expression was significantly inhibited in MSCs derived from CS patients.

Subsequently, the total glutathione amount as well as the main glutathione dependent enzymes’ activities were analyzed. In summary, the amount of total glutathione as well as the enzymatic activities were up-regulated in IATRO-MSCs when compared to the others, whereas C-MSCs and ENDO-MSCs displayed almost the similar amounts, and the administration of steroid sparing agents contributed to preserve the global antioxidant capacity. Glutathione (GSH) is a ubiquitous intracellular peptide involved in detoxification, antioxidant defense, maintenance of thiol status, and others (Armeni et al., 2012, 2014; Scirè et al., 2019).

Our data showed an increase of glutathione levels and an up-regulation of GCS in IATRO-MSCs compared to the C-MSCs. These results are in line with others: Lu (2013) reported an increase in the expression of GLC by 45–65% (earliest significant change at 4 h) but not in GSH synthetase, with an increase of GSH up to 50–70% in cultured hepatocytes treated with hydrocortisone (HC, 50 nM).

Glutathione homeostasis in the cell is not only regulated by its *de novo* synthesis, but also by other factors such as utilization, recycling, and cellular export. This redox cycle is known as the GSH cycle and incorporates other important antioxidant, redox-related enzymes (Armeni et al., 1997).

The observations that all these molecule/enzymes reached the highest level in MSCs isolated from skin of patients with iatrogenic CS reflect different abilities across the cells types to counteract the oxidative stress. IATRO-MSCs are still able to counteract the excess of exogenous GCs whereas MSCs from endogenous CS, normally experienced to elevated exposure to GCs, have developed mechanisms of adaptation. When steroid sparing agents were administered, the level of glutathione decreased and, in turn, the main related enzymes reduced their activity. These results suggest that the defense mechanisms against oxidative stress were still responsive and well-functioning in iatrogenic CS patients treated with steroid sparing agents.

The analysis of the expression of the related genes gave different results: ENDO-MSCs expressed higher amounts of genes than the other cell groups. This discrepancy is only apparent; it has been previously reported in different models, ranging from vegetables up to marine organisms, and mammals (Regoli and Giuliani, 2014; Yang et al., 2014), that the relationships occurring between transcriptional and catalytic antioxidant responses are too complex to hypothesize a direct effect between mRNA and enzyme activity. Discrepancies in the responses of different antioxidants are linked to asynchronous activation, displaying different time-courses of activation both at the transcriptional and catalytic levels. Great attention should also be focused on chronic exposures to GCs in endogenous CS patients. During chronic exposure, antioxidant defense mechanisms develop adaptive and compensatory mechanisms: at the beginning, the excess of GCs is counteracted by the induction of the enzymes that progressively decrease, up to the depletion of their activity.

In conclusion, the excess of GCs observed in endogenous and exogenous CS affects the behavior of MSCs that show the hallmarks of CS, such as the same cytokines altered expression observed in course of wound healing impairment, detrimental differentiative potential, and dysregulated anti-oxidant systems, allowing in fact backdate the onset of this pathology at the level of MSC.

## Data Availability Statement

All data generated or analyzed during this study are included in this published article (and its supplementary information files).

## Ethics Statement

The studies involving human participants were reviewed and approved by institutional ethics committees (CERM), 2016-0360OR. The patients/participants provided their written informed consent to participate in this study.

## Author Contributions

MC isolated and characterized the MSCs. TA performed the enzymatic analysis. PP and LC performed the molecular analysis. MM and GD enrolled the patients. AO contributed to analysis of the results. GA, AC, and MO projected the research, analyzed the results, and wrote the manuscript. All authors read and approved the final manuscript.

## Conflict of Interest

The authors declare that the research was conducted in the absence of any commercial or financial relationships that could be construed as a potential conflict of interest.
